# Grading Central Diabetes Insipidus Induced by Immune Checkpoint Inhibitors: A Challenging Task

**DOI:** 10.3389/fendo.2022.840971

**Published:** 2022-03-21

**Authors:** Agnese Barnabei, Lidia Strigari, Andrea Corsello, Rosa Maria Paragliola, Giovanni Maria Iannantuono, Roberto Salvatori, Salvatore Maria Corsello, Francesco Torino

**Affiliations:** ^1^ Endocrinology Unit, Presidio Ospedaliero Santo Spirito in Sassia, Azienda Sanitaria Locale Roma 1, Rome, Italy; ^2^ Medical Physics Department, Istituto di Ricovero e Cura a Carattere Scientifico (IRCCS) Azienda Ospedaliero-Universitaria di Bologna, Bologna, Italy; ^3^ Department of Translational Medicine and Surgery, Unit of Endocrinology, Università Cattolica del Sacro Cuore-Fondazione Policlinico “Gemelli” Istituto di Ricovero e Cura a Carattere Scientifico (IRCCS), Rome, Italy; ^4^ Department of Systems Medicine, Medical Oncology, Tor Vergata University of Rome, Rome, Italy; ^5^ Division of Endocrinology, Diabetes, and Metabolism and Pituitary Center, Johns Hopkins School of Medicine, Baltimore, MD, United States; ^6^ UniCamillus Chair of Endocrinology, Saint Camillus International University of Health Sciences, Rome, Italy

**Keywords:** central diabetes insipidus, immune checkpoint inhibitors, grading system, CTCAE, endocrine toxicities

## Abstract

Central diabetes insipidus (CDI) is a rare endocrine disease deriving from an insufficient production or secretion of anti-diuretic hormone. Recently, CDI has been reported as a rare side effect triggered by immune checkpoint inhibitors (ICI) in cancer patients. Despite its current rarity, CDI triggered by ICI is expected to affect an increasing number of patients because of the expanding use of these effective drugs in a growing number of solid and hematologic malignancies. An appropriate assessment of the severity of adverse events induced by anticancer agents is crucial in their management, including dosing adjustment and temporary withdrawal or discontinuation treatment. However, assessment of the severity of CDI induced by ICI may be challenging, as its main signs and symptoms (polyuria, dehydration, weight loss, and hypernatremia) can be incompletely graded. Indeed, the current grading system of toxicity induced by anticancer treatments does not include polyuria. Additionally, dehydration in patients affected by diabetes insipidus, including ICI-induced CDI, is different in certain aspects from that due to other conditions seen in cancer patients, such as vomiting and diarrhea. This prompted us to reflect on the need to grade polyuria, and how to grade it, and to consider a specific grading system for dehydration associated with CDI induced by ICI. Here we propose a new grading system for polyuria and dehydration, as critical symptoms of the CDI syndrome occurring in patients on ICI treatment, to obtain better management of both the adverse event and the triggering drugs.

## Introduction

Anti-diuretic hormone (ADH) (also called Vasopressin) is produced by hypothalamic supraoptic and paraventricular nuclei, stored and secreted at the level of the posterior pituitary. ADH is initially synthesized as a pro-hormone (pre-pro-ADH) that is cleaved generating a 9-amino-acid hormone (ADH) and equimolar amounts of a more stable C-terminus peptide called copeptin. ADH causes water reabsorption through the V2 receptor-mediated insertion of aquaporin water channels into the luminal membrane in the collecting duct of the kidney (1). Diabetes insipidus (DI) manifests when a decreased activity of ADH occurs. It can be due to partial or complete secretion failure (central DI, CDI), or to renal resistance to its effect (nephrogenic DI, NDI) (2). CDI may derive from injury to the pituitary or the hypothalamus, while NDI is due to insensitivity of the kidney receptor to ADH. The leading causes of CDI and NDI are reported in **Table 1**. The work-up for CDI diagnosis in cancer patients follows the recognition of critical early symptoms, including polyuria, nocturia, excessive thirst, polydipsia, dehydration, weight loss, lethargy, and confusion (44, 45). Once CDI is suspected, endocrinological consultation is recommended (2). As the first diagnostic step, the presence of hypotonic polyuria should be confirmed, then the type of polyuria-polydipsia disorder (central DI vs. nephrogenic DI vs. primary polydipsia) should be identified (44, 45). To this aim, the endocrinologist will opt either to require the water deprivation test (WDT) or the hypertonic saline infusion test, along with serum copeptin measurements (45). Once the disorder is recognized, the underlying etiology needs to be identified among several potential causes, based on an accurate medical history and choosing the appropriate biochemical and imaging tests (44, 45).

**Table 1:** Causes of central (injury to the pituitary and/or hypothalamus) and nephrogenic diabetes insipidus.

	Central diabetes insipidus	Nephrogenic diabetes insipidus
Autoimmune/ Inflammatory (2–6)	•Lymphocytic hypophysitis •Xanthogranulomatous hypophysitis •IgG4 disease •Anti-vasopressin neuron antibodies •Guillain-Barré syndrome	—
Congenital (genetic) (1, 7–12)	•AVP-neurophysin II gene alterations •Wolfram (DIDMOAD) syndrome •Septo-optic dysplasia •Schinzel-Giedion syndrome •Culler-Jones syndrome •Alstrom syndrome •Hartsfield syndrome •Webb-Dattani syndrome •X-linked defects with subnormal AVP levels	•Aquaporin-2 channel gene alterations •X-linked V-2 receptor gene alterations •PMSE syndrome (polyhydramnios, megalencephaly, and symptomatic epilepsy) •Type 4b Bartter syndrome
Drugs/toxins (4, 13–29)	•Temozolomide •Immune checkpoint inhibitors •Phenytoin •Ethyl alcohol, snake venom	•Lithium •Demeclocycline, Methoxyflurane •Cisplatin, pemetrexed •Aminoglycosides, amphotericin B
Granulomatous or systemic disease (4, 30–33)	•Sarcoidosis •Granulomatous hypophysitis •Langerhans’ cell histiocytosis •Erdheim-Chester disease	•Amyloidosis •Sarcoidosis •Sjogren’s syndrome
Infectious (2–4, 34)	•Meningitis, encephalitis •Tuberculosis •Pituitary or hypothalamic abscess	—
Neoplastic (4, 35–37)	•Craniopharyngioma, germinoma, meningioma •Invasive pituitary macroadenoma •Pituitary and/or hypothalamus metastasis	•Multiple myeloma
Trauma (38–42)	•Deceleration injury •Intracranial surgery •Transsphenoidal pituitary surgery	—
Vascular (4, 43)	•Hypothalamic infarction/hemorrhage •Cerebral infarction/hemorrhage •Anterior communicating artery ligation/aneurysm •Sheehan’s syndrome •Sickle cell disease	•Renal infarction •Sickle cell disease
Renal disease (1–4)	—	•Chronic kidney disease •Polycystic kidney disease •Obstructive uropathy
Metabolic (1–4)	—	•Hypokalemia •Hypercalcemia

The consequence of DI is a variably decreased ability to concentrate urine, leading to polyuria and polydipsia. However, polyuria (arbitrarily defined as a urine volume >3 Liters/day or ≥50 ml/Kg/24 hours) is considered the hallmark of DI and may arise suddenly in CDI, being usually more insidious in NDI (44). The grade of polyuria severity depends on the total solute load, the circulating volume, and the DI severity. Nocturia may be the main symptom in mild DI and the first clue to its diagnosis (2, 44). When DI derives from an injury to the hypothalamus-pituitary (CDI), it may be accompanied by deficiency of anterior pituitary hormones such as adrenocorticotropic hormone (ACTH), resulting in adrenal insufficiency, TSH in central hypothyroidism, gonadotropins in hypogonadism, and deficit of growth hormone and prolactin (3, 44). Notably, in mild CDI, polyuria may not be revealed until the adrenal insufficiency is treated since cortisol deficiency increases fluid reabsorption and ADH release and reduces glomerular filtration rate (44). When thirst mechanisms are intact, and access to water is accessible, DI does not result in dehydration and overt hypernatremia (defined as a serum Na >145 mEq/L) (2, 3, 44, 45). Conversely, if thirst or access to water (or both) is somewhat impaired, the persistence of polyuria may cause fluid depletion, leading to hypernatremia and a rapid weight loss (2, 44). This, in turn, may also reduce the effective circulating volume (hypovolemia), causing impairment of tissue and organ perfusion. If severe hypovolemia is not timely corrected, ischemic end-organ damage occurs, leading to life-threatening conditions, up to death if patients are in shock (or affected by other severe comorbidities) (46). Unrecognized or new-onset DI leading to symptomatic hypernatremia in a patient with altered mental status, impaired thirst mechanism, or restricted access to water, may become an emergency condition. In particular, hypothalamic disorders (e.g., tumors, granulomatous disorders, and vascular disease) can result in both DI and impaired thirst sensation (“adipsic DI”) (2, 44, 46). Notably, cancer patients may not suffer from any of those conditions, but they may reduce their fluid intake due to nausea, vomiting, fatigue, and malaise, symptoms frequently caused by anticancer treatments and malignancy itself. These conditions may hamper compensating hypernatremia by drinking, leading to a rapid and potentially severe worsening of DI.

Herein, we focused on CDI in cancer patients on treatment with immune checkpoint inhibitors (ICI) and the hurdles of assessing its severity in this subgroup of patients.

## CDI in Cancer Patients

In cancer patients, CDI may arise when local malignancies or metastases compress or infiltrate the posterior pituitary or the supraoptic/paraventricular nuclei of the hypothalamus, or when the function of these structures is impaired by anticancer treatments, such as brain surgery and/or radiotherapy. CDI is rarely diagnosed as a paraneoplastic syndrome (3, 47) or as a side effect of certain anticancer drugs (i.e., temozolomide) (13–16). In recent years, CDI has been reported in a limited number of cancer patients on ICI (17–28). Three classes of ICI are currently available in the clinic: anti-CTLA4 monoclonal antibodies (anti-CTLA4 mAb) and monoclonal antibodies targeting the programmed cell death receptor-1 (PD-1) or its ligands (PD-L1) (anti-PD1 mAb and anti-PDL1 mAb) (48). ICI have demonstrated improvements in survival in patients affected by several malignancies, and their use is expected to increase in the near future with further indications and new agents. ICI act by restoring the immune competence against cancer cells after escaping the control of the immune system (49). However, ICI may trigger several autoimmunity/autoinflammatory adverse events (irAEs) intimately related to their mechanism of action, i.e., the selective stimulation of the host immune system (48, 50). Endocrine irAEs are among the most frequent ICI-related toxicities, being thyroid and pituitary dysfunction prevalent (51–53).

## CDI in Patients on Treatment With ICI

CDI induced by ICI is a rare endocrine irAE. Bai et al., in the WHO global database of individual case safety reports (54), in the period between January 2011 and March 2019, found a total of 6,089 ICI-related endocrine side effects. Out of these side effects, 1,144 (18.8%) were pituitary events, including hypophysitis (835 reports), hypopituitarism (268 reports), pituitary enlargement (52), other (18), while CDI was reported in 7 out of 1,072 (0.7%) of the registered hypophysitis/hypopituitarism cases. Recently, we systematically reviewed the literature and found eleven papers reporting on patients who suffered from ICI-induced CDI (Barnabei et al., accepted manuscript; in press). In five of those cases, CDI was diagnosed in the context of a panhypophysitis induced by ipilimumab (an anti-CTLA4 mAb): in three of them, ipilimumab was administered as a single agent (18–20), while in the other two cases, ipilimumab was administered in combination with nivolumab (an anti-PD1 mAb) (21, 22). In four of the 11 cases, CDI was diagnosed as an isolated endocrine irAE: the triggering drug was either avelumab (an anti-PDL1 mAb) (23), nivolumab (an anti-PD1 mAb) (24), or sintilimab (an anti-PD1 mAb) (25), while in the fourth case, CDI was reported in a patient who received a combination treatment (tremelimumab + durvalumab, an anti-CTLA4 mA and an anti-PDL1 mAb, respectively) (26). In another case, CDI occurred in the context of hypothalamitis caused by atezolizumab (an anti-PDL1 mAb) (28). In the last case, CDI was reported in a patient on nivolumab, diagnosed with a concomitant anterior pituitary metastasis (27). The analysis of those case reports did not provide unifying clinical features of the ICI-induced CDI syndrome. Indeed, the work-up that led to the diagnosis and even terms used to describe the CDI syndrome varied. Obviously, once CDI was diagnosed, therapy with vasopressin or its longer acting analog de-amino D-arginine vasopressin (DDAVP or desmopressin) was rapidly started in most cases, and compensation was obtained, but information about how long replacement therapy was continued is unavailable in many reports. Also, the management of the causal drug(s) varied, as in two cases, the anticancer treatment was transiently stopped, in two cases completed, while in the other 9 cases, ICI was permanently discontinued. However, the reasons leading to either maintenance or withdrawal of the triggering ICI(s) were based on clinical judgment, in the absence of specific guidelines for the management of CDI as an irAE induced by ICI.

Studies exploring the pathogenic mechanisms leading to the onset of CDI in patients on ICIs are currently unavailable. It is speculated that autoimmunity triggered by these drugs might impair the anterior pituitary leading to the inflammatory damage of the posterior pituitary or both (51, 55–57). The hypothesis reflects the pathogenesis of other ICI-induced organ damage, including thyroid and other endocrine glands (58, 59). Interestingly, selective injury to the posterior pituitary or the hypothalamus has been suggested. Specifically, the expression of the PD-L1 on hypothalamic cells of a primate has been recently demonstrated (60), providing the basis for a potential explanation for the onset of hypothalamitis that occurred during treatment with atezolizumab (28). Histological data would be essential in clarifying the pathogenesis of ICI-induced CDI; however, biopsy specimens are difficult to obtain for various reasons, including the unethicality of the procedure in certain clinical conditions. Therefore, studies exploiting autoimmunity antibodies in this subgroup of patients would be essential (61–64).

As in other rare irAEs triggered by ICI (65), with the growing clinical use of these agents, a better knowledge of the CDI syndrome induced by ICI may help oncologists early suspect its onset and early activation endocrinologist consultation. Moreover, a specific grading system capable of adequately assessing the severity of CDI as an irAE triggered by ICI would be helpful in the choice of maintaining, delaying, or withdrawing the causative drug(s). However, some hurdles need to be overcome.

## Emerging Problems in Grading ICI-Induced CDI

Anticancer drugs have a narrow therapeutic range. Therefore, their starting dose is carefully assessed in clinical practice, based on the drug schedule, patient’s parameters (i.e., performance status, comorbidities, age, organ function impairments, etc.), and, when available, pharmacogenetic factors predicting toxicity (i.e., polymorphisms of dihydropyrimidine dehydrogenase gene if fluoropyrimidines will be used, etc.) (63, 64, 66, 67). After that, the management of anticancer drugs includes the severity of adverse events (level of toxicity) reported after each administration, measured according to the Common Terminology Criteria for Adverse Events (CTCAE) (68). In detail, CTCAE is an updated list of terms describing adverse events (AE) commonly encountered in oncology practice and research, intended to be an agreed-on terminology for the designation, reporting, and grading of AE. Each term indicating an AE is defined, and the severity of AE is classified according to a 5-level scale corresponding to increasing levels of severity (from mild, categorized as grade 1, to patient’s death due to toxicity, categorized as grade 5). Laboratory parameters or clinical features are used to grade the severity of each AE (examples in **Table 2**). The CTCAE grading system, through the objective assessment of toxicity experienced by single patients at each treatment administration, informs clinicians if dose adjustments to the treatment plan are needed (CTCAE). Consistently, the management of ICI is based on the level of the reported toxicity, assessed according to the current CTCAE grading system, recently updated including irAEs. Based on CTCAE assessment, the major scientific societies (e.g., ESMO, ASCO, SITC, NCCN) provided detailed guidelines for managing ICI-related toxicities, including endocrine irAEs (69–72). However, recommendations for the management of the causal drug(s) in patients diagnosed with ICI-induced CDI are not yet available, presumably due to the rarity of this irAE. Only the NCCN guidelines suggest “considering workup for diabetes insipidus if a patient complains of polyuria/polydipsia and elevated natremia” (NCCN). However, as CDI is emerging as a new irAe induced by ICI, a reflection on the potential hurdles in assessing the severity of the related symptoms has appeared as timely.

**Table 2:** Toxicity level of the main symptoms (dehydration, hypernatremia, weight loss) of diabetes insipidus according to the current CTCAE grading system (version 5.0).

	Grade 1	Grade 2	Grade 3	Grade 4	Grade 5
**Dehydration**	Increased oral fluids indicated; dry mucous membranes; diminished skin turgor	IV fluids indicated	Hospitalization indicated	Life-threatening consequences; urgent intervention indicated	Death
Definition: A disorder characterized by excessive loss of water from the body. It is usually caused by severe diarrhea, vomiting or diaphoresis.					
**Hypernatremia**	>ULN - 150 mmol/L	>150 - 155 mmol/L; intervention initiated	>155 - 160 mmol/L; hospitalization indicated	>160 mmol/L; life-threatening consequences	Death
Definition: A disorder characterized by laboratory test results that indicate an elevation in the concentration of sodium in the blood.					
**Weight loss**	5 to <10% from baseline; intervention not indicated	10 - <20% from baseline; nutritional support indicated	>=20% from baseline; tube feeding or TPN indicated	–	–
Definition: A finding characterized by a decrease in overall body weight; for pediatrics, less than the baseline growth curve.					
**Urinary frequency**	Present	Limiting instrumental ADL; medical management indicated	–	–	–
Definition: A disorder characterized by urination at short intervals					

ADL, activities of daily living; IV, intravenous; TPN, total parenteral nutrition; ULN, upper limits of normal values.

It is widely agreed that polyuria is the hallmark of DI. Similarly, it is well known that polyuria in DI, if not compensated (by adequate fluid intake or vasopressin or desmopressin), may lead to hypernatremia, dehydration, and weight loss. Indeed, the ADH deficit is responsible for pure water loss, leading to elevation in serum osmolality and sodium concentration and, therefore, to the passage of water from the cells into extracellular fluid (due to an osmotic gradient) (3, 46, 47). As in DI approximately two-thirds of the pure water loss derives from the intracellular fluid, the condition is more appropriately defined as “dehydration” than “hypovolemia” (46). Importantly, patients with pure water loss display the symptoms of hypernatremia (produced by the water deficit) before those of marked extracellular fluid depletion (46). Therefore, the assessment of polyuria, dehydration, and hypernatremia are the three critical components of the DI syndrome to consider in evaluating CDI severity. However, in the current CTCAE grading system hypernatremia and dehydration are graded, but not polyuria (**Table 2**) (68). The closer condition to polyuria categorized in the CTCAE grading system could be “urinary frequency”, defined as “a disorder characterized by urination at short intervals” (68). Grade 1 urinary frequency is determined as “present”, while grade 2 occurs when urinary frequency limits instrumental ADL and/or medical management is/are indicated (**Table 2**). However, according to its definition, urinary frequency is a synonym of pollakiuria, which describes the frequent elimination of normal volumes of daily urine, while polyuria indicates the urination of larger than normal urine volume. It could be speculated that grading polyuria could not be necessary to assess the severity of ICI-induced CDI, as hypernatremia and dehydration provide enough information about the seriousness of DI. However, in patients affected by CDI, normal natremia or mild hypernatremia, like mild dehydration, may not be informative about the severity of the condition, as patients may compensate through a variably high intake fluid. Notably, the compensation obtained by drinking could mask the severity of DI if polyuria (and polydipsia) are not considered in the evaluation. In other words, the patient on ICI who develops CDI could present with mild hypernatremia or even normal values of natremia and/or mild (or no) dehydration, at the cost of an increased fluid intake. Importantly, the patient’s conditions may rapidly worsen if not adequately diagnosed and assessed. Therefore, polyuria seems to be the key symptom to evaluate not only to obtain an early diagnosis of CDI but also to estimate the severity of ICI-induced CDI (essential in the further management of ICI).

Moreover, other symptoms are reported among those that may worsen the “day and night” quality of life of patients affected by DI (e.g., thirst and the compelling need for quick water drinking, urinary frequency, nocturia, and the quality of sleeping, etc.). These symptoms should also be considered in the comprehensive evaluation of ICI-induced CDI and its management.

Finally, the assessment of dehydration related to DI needs careful considerations in cancer patients. In the current CTCAE grading system, the assessment of dehydration severity is not quantitative, based on the need for fluid supplementation and the level of assistance required by the patient (**Table 2**) (68). This is a proper evaluation in patients presenting with loss of fluids due to diarrhea and vomiting. Notably, diarrhea and vomiting, even in their severe forms, may lead to weight loss in a longer time compared with the “rapid” (in a few hours) weight loss induced by polyuria due to DI. This highlights the need for a proper grading system for dehydration in the context of DI, which would be helpful particularly when dehydration occurs in patients with CDI induced by ICI.

## Can ICI-Induced CDI Be Better Assessed?

In our opinion, the assessment of ICI-induced CDI severity would improve if a quantitative evaluation of both polyuria and dehydration/weight loss is considered. In the literature, approaches considering the quantitative assessment of these symptoms are available.

Vedig (73) identified different severity levels of polyuria based on the loss of urine volume/body weight unit/hour and arbitrarily classified polyuria into two grades: mild (<3ml/kg/h) and severe (>7 ml/kg/h for 4-6 h). To respect the standard CTCAE setting, where toxicities are classified into five severity levels, we suggest maintaining the two grades as proposed by Vedig (i.e., mild = grade 1 and severe = grade 3), adding both the grade 2 level, corresponding to moderate polyuria (3-7 ml/kg/h) and grade 4, corresponding to a life-threatening condition (**Table 2**). In the last level, patients with any grade polyuria associated with moderate-severe dehydration and/or moderate-severe hypernatremia should be included (while level 5 toxicity will remain the case of patient death due to treatment toxicity).

Regarding dehydration, it should be noted that the term is often used interchangeably with volume depletion/hypovolemia to indicate a reduction in the circulating volume because of vomiting, diarrhea, diuretics, bleedings, and polyuria as occurring in DI. To better classify the weight loss induced by polyuria due to DI, we considered the yardstick criteria of WDT, and the classification created to define weight loss in the pediatric setting. In infants and children, a quantitative approach is used to assess dehydration based on evaluating signs and symptoms related to volume depletion (74). Hypovolemia is divided into three grades: mild (corresponding to 3-5% volume loss), moderate (6-9% volume loss), and severe (≥10% volume loss). With this premise, to harmonize the scale as mentioned earlier with the standard 5-grade classification used in the CTCAE, we propose to adapt this classification of dehydration to the CTCAE setting by adding the level “mild”, indicating a volume/weight loss <3% to the other toxicity levels (considering 3-5% volume loss as moderate, i.e., G2; 6-9% volume loss as sever, i.e., G3; ≥10% volume/weight loss or a shock condition as life-threatening, i.e., G4 (G5 defining death occurring due to treatment toxicity) (**Table 3**). Additionally, considering the time frame in which dehydration ensues may further improve the assessment. This is because dehydration due to loss of water (weight loss) occurring in moderate-severe DI is typically more rapid compared to that caused by most other conditions (e.g., diarrhea or vomiting). Therefore, we suggest grading the severity of polyuria-induced dehydration in patients developing ICI-induced CDI, considering weight loss/unit of time (another quantitative assessment) (**Table 3**). This derives from the fact that weight loss/unit of time is used in the WDT, an essential tool in diagnosing DI. WDT measures the capacity of the kidney to concentrate urine in response to dehydration. It can also assess kidney response to desmopressin, verifying if replacement with desmopressin can correct the defect identified in urine concentrating ability. Weight, urine volume, and serum and urine osmolality are measured at baseline and every two hours along with the test. WDT ends if thirst becomes unbearable or if the patient loses >5% initial weight, as measured at each unit of time (two hours) (2, 44, 45). Notably, in older patient excessive fluid loss often presents with nonspecific signs and symptoms, being acute weight loss the most specific sign for hypovolemia. As there is less water in fat than muscle, older individuals have lower total body water (relative to weight). Consequently, for a given degree of fluid loss, those individuals will have a more significant reduction in extracellular fluid volume. Therefore, acute fluid loss reflects body weight loss, so that a two-liter of fluid loss corresponds to two-kilogram weight loss (46).

**Table 3:** Suggested classification of polyuria and dehydration in the ICI-induced CDI syndrome.

Grade	1 (mild)	2 (moderate)	3 (severe)	4 (life-threatening)	5
Polyuria	<3 ml/kg/h	3÷7 ml/kg/h	>7 ml/kg/h	Any grade polyuria + moderate-severe dehydration ± moderate-severe hypernatremia	Death
Dehydration	Loss of <3% body weight in 2 hours	Loss of 3 ÷ 5% body weight in 2 hours	Loss of 6 ÷ 9% body weight in 2 hours	Loss of ≥10% body weight in 2 hours or shock	Death

## Practical Management of Patients Diagnosed With ICI-Induced CDI

**Figure 1:**
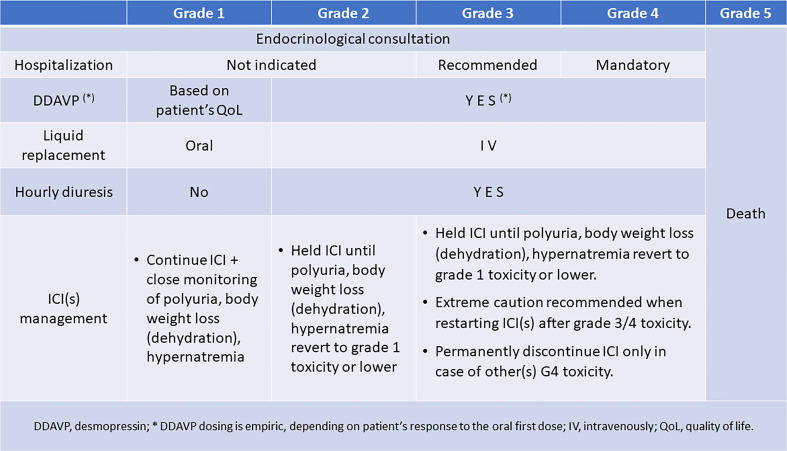
The suggested management of desmopressin and ICI(s) in patients with ICI-induced CDI.

Specific guidelines to properly manage patients who develop ICI-induced CDI are urgently needed. Meanwhile, we suggested managing ICI-induced CDI within a multidisciplinary team, including oncologists and endocrinologists (**Figure 1**). Endocrinology consultation should be required early, as soon as the patients or their caregivers report the onset of polyuria and polydipsia. In case of mild (grade 1 or moderate (grade 2) toxicity, patient hospitalization is not indicated, being recommended in case of grade 3 toxicity and mandatory in case of grade 4 toxicity. Replacement therapy is always indicated in the case of grade 2-4 toxicity, while in grade 1 toxicity, it should be considered by the endocrinologist based on the impact of symptoms (mainly polyuria) on the patient’s quality of life. Fluid replacement can be obtained by oral intake in grade 1 polyuria, dehydration/weight loss, or hypernatremia. While vasopressin must be administered parenterally and has a short duration of action (2-8 hours), desmopressin’s effect is longer (6-9 hours and possibly longer, often allowing for twice a day administration) and it can also be administered intranasally, sublingually, or orally (75). Oral and sublingual absorption rates are <1%, whereas intranasal is approximately 6% (76). The mean dose ratio of sublingual to intranasal DDAVP is 1:24 (77). Physicians should be familial with different modalities of ADH replacement, their duration of action and equivalencies when transitioning from one therapy to another one. Intravenous fluid replacement becomes recommended when toxicities are graded as 2-4, together with an hourly diuresis monitoring. Finally, in the case of ICI-induced CDI, the causal agent should not be withdrawn unless other life-threatening irAEs have been experienced or persist. This approach is commonly recommended in patients presenting with other endocrine irAEs (69–72). However, it should be noticed that, independently of the severity of CDI symptoms, the ICI administration should be delayed to when the toxicity lessens to G1 (mild) level or symptom(s) disappear, indicating a compensation of the dysfunction. The delay allows testing the efficacy of vasopressin or desmopressin and its dose titration in every patient. This is in the perspective of restarting ICI(s) as soon as clinically indicated, considering the need for cancer control. Importantly, patients on ICI and their caregivers should receive clear information on the importance of alerting the reference care team at the onset of polyuria, polydipsia, and weight loss to timely obtain the appropriate diagnostic work-up and treatment.

## Conclusions

CDI is a rare side effect triggered by ICI, but with the expanding use of these effective drugs, it is expected to be increasingly diagnosed in cancer patients. The current assessment of the severity of ICI-induced CDI may be challenging. We suggested a new grading system of polyuria and dehydration, as critical symptoms of the CDI syndrome occurring in patients on ICI treatment, to obtain better management of both the adverse event and the triggering drugs. Our proposals are attempts to overcome the emerging hurdles in assessing ICI-induced CDI severity. Studies are ongoing to define the reliability of the suggested classifications in clinical practice. At the moment, the evaluation of the severity of ICI-induced CDI should be only based on dehydration and hypernatremia levels, assessed by using the current CTCAE grading system, while the management of patients and ICI(s) treatment should still be based on a case-by-case approach in a multidisciplinary team.

## Author Contributions

Conceptualization, AB, LS, RS, SC, and FT. Investigation, AB, AC, RP, GI, and FT. Data curation, AB, LS, RS, GI, SC, and FT. Writing—original draft preparation, AB, LS, RS, SC, and FT. Writing—review and editing, AB, LS, RS, SC, and FT. Visualization, AB, LS, GI, and FT. Supervision, AB, SC, and FT. All authors have read and agreed to the published version of the manuscript.

## Conflict of Interest

The authors declare that the research was conducted in the absence of any commercial or financial relationships that could be construed as a potential conflict of interest.

## Publisher’s Note

All claims expressed in this article are solely those of the authors and do not necessarily represent those of their affiliated organizations, or those of the publisher, the editors and the reviewers. Any product that may be evaluated in this article, or claim that may be made by its manufacturer, is not guaranteed or endorsed by the publisher.
